# Zeolitic Imidazolate Framework‐8 as pH‐Sensitive Nanocarrier for “Arsenic Trioxide” Drug Delivery

**DOI:** 10.1002/chem.201902599

**Published:** 2019-09-13

**Authors:** Romy Ettlinger, Natalia Moreno, Dirk Volkmer, Kornelius Kerl, Hana Bunzen

**Affiliations:** ^1^ Chair of Solid State and Materials Chemistry Institute of Physics University of Augsburg Universitaetsstraße 1 86159 Augsburg Germany; ^2^ Pediatric Hematology and Oncology University Children's Hospital Muenster Albert-Schweitzer-Campus 1 48149 Muenster Germany

**Keywords:** arsenic, cancer, drug delivery, metal–organic frameworks, nanoparticle

## Abstract

Previous results revealed that arsenic trioxide might be used as promising therapeutic agent for the treatment of some solid tumours as atypical teratoid rhabdoid tumours (ATRT). However, in order to become an approved drug for solid tumour treatment, the active formulation has to get more efficient and feasible—but at the same time less toxic. One of the possibilities to achieve this dichotomy is to use nanomedicine tools. Herein, we report on the Zn‐based metal–organic framework ZIF‐8 (*Z*eolitic *I*midazolate *F*ramework‐8) which turned out to be a promising candidate for the delivery of As^III^ species. It conjointly features a high drug loading capacity and a prominent pH‐triggered release behaviour. As^III^‐loaded ZIF‐8 nanoparticles coated and non‐coated with polyethylene glycol were studied by XRPD, IR, Raman, TGA, TEM, EDX, CHN‐elemental analysis, sorption analysis and ICP‐OES, and their cytotoxicity was evaluated in vitro.

## Graphical abstract

**Studies showed that ZIF‐8** nanoparticles released only a little As^III^‐drug at the neutral pH, whilst a complete drug release took place at the more acidic pH value. In vitro cytotoxicity tests revealed that the loaded drug increased the material cytotoxicity against cancer cells, whilst not influencing the toxicity against fibroblast.

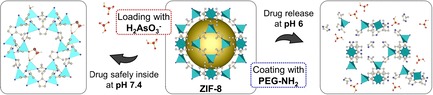

## Introduction

In 2000 arsenic trioxide (ATO) was approved by the FDA (US Food and Drug Administration) as a drug for treating refractory or relapsed acute promyelocytic leukaemia (APL)^1^ and since 2016 it is also approved by the EMA (European Medicines Agency) for treating newly diagnosed APL.^2^ Expending the medical use of ATO is challenging and often fails due to its high toxicity—only 15 mg kg^−1^ (lethal dose, rat, oral).^3^ We recently reported on using ATO in the treatment of atypical teratoid rhabdoid tumours (ATRT).^4^, ^5^ ATRT is a brain tumour entity, which occurs principally in young children under the age of three years and has a very poor prognosis despite the use of intensive and multimodal treatment. In our report, we showed that ATO is a promising drug to treat this aggressive paediatric tumour entity. However, reaching the targeted tissue by ATO in therapeutic levels is challenging. Here, nanomedicine could play an important role.

Using nanocarriers for drug delivery has been considered to be effective in suppressing negative systematic side effects of the distributed drugs, while retaining their therapeutic effects.^6^, ^7^ Several nanocarriers based on various materials have been reported in literature for delivery of ATO. These include polylactic acid/magnetic hybrid nanoparticles, polyacrylic acid capped mesoporous silica nanoparticles (MSNs) and magnetite doped mesoporous silica nanoparticles. They have been used to improve the therapeutic effects of arsenic species in cancer therapy.^8^, ^9^, ^10^ Despite the reported positive results of using nanocarriers for ATO delivery, the amounts of the carried drug were not too high with respect to the amount of the carrier materials used (see Table 1). Moreover, the ATO release from these nanocarriers was not well‐defined triggered or complete. To improve the loading capacity and the release behaviour, we decided to use porous materials known as metal–organic frameworks (MOFs). MOFs are crystalline materials composed of organic ligands and metal ions or clusters, which provide a huge variety of outstanding functional properties,^11^, ^12^ which enable versatile applications as gas storage and separation,^13^, ^14^ catalysis,^15^, ^16^ sensing^17^, ^18^ and many others.^19^ The well‐defined pores and exceptionally high internal surface areas make them perfect candidates for drug carrier materials, because large amounts of drug molecules can be accommodated inside the pores within the whole material volume. Recently we reported on a proof‐of‐principle study of using a MOF called MFU‐4l as a carrier material of arsenic drugs.^20^ We showed that it was possible to bind an arsenic drug to a MOF, in order to achieve a high loading (equivalent of 237 μg of As_2_O_3_/1 mg, Table 1), while keeping the therapeutic effects of the drug. On our systematic research for other suited MOF nanocarriers, we discovered that a Zn‐based metal–organic framework called ZIF‐8 (*Z*eolitic *I*midazolate *F*ramework) could be an even more promising candidate than the reported MFU‐4l. ZIF‐8 could not only enable a high drug loading capacity due to its high porosity but possibly also a pH‐triggered drug release due to the Zn−N coordinate bond being less stable in acidic conditions.^21^, ^22^ This possibility of triggered release could be an important add‐on to the material properties in order to prepare a drug carrier for targeted drug delivery.

**Table 1:** Selected nanocarriers of ATO reported in literature.

Nanocarrier	As_2_O_3_ loading (μg per 1 mg)
polylactic acid/magnetic hybrid nanoparticles^8^	78–139
polyacrylic acid capped MSNs^9^	35
magnetite doped MSNs^10^	111
metal–organic framework MFU‐4l^20^ (not pH‐responsive)	237
metal–organic framework ZIF‐8^[this work]^ (pH responsive)	98

Nowadays, one of the goals in nanomedicine is to achieve targeted drug delivery, which would mean that a drug is selectively delivered only to the desired place within the body without affecting the rest of the tissues.^23^ One possibility would be to develop drugs or drug nanocarriers decorated with efficient targeting ligands.^24^ Another strategy is to develop carriers which might not be able of selective delivery, but are able of selective drug release, that is, while being distributed within different parts of the body, the nanocarrier keeps the drug safely inside, and thus, protects the body until the drug is delivered to a specific side, where the drug release is triggered.^25^ In cancer therapy, for instance, a change in pH could be used as a trigger.^26^ Due to the high metabolic rate of cancer cells, the extracellular microenvironment of cancer tissues tend to be more acidic than the remaining tissues.^27^ Thus, a carrier with drug release triggerable by a pH change from neutral to slightly acidic would be very desirable in cancer treatment. Therefore, the aim of this work was to develop a pH‐responsive nanocarrier of arsenic trioxide based on a metal–organic framework, and to study its drug release kinetics at different pH values and evaluate its cytotoxicity.

## Results and Discussion

### Nanoparticle synthesis and drug loading

**Figure 1:**
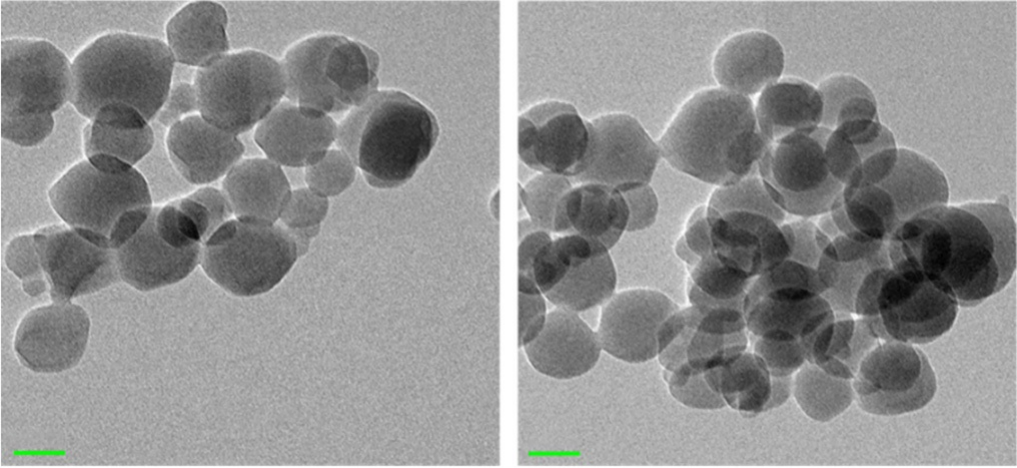
TEM image of ZIF‐8 nanoparticles before (left) and after the As‐drug loading, scale bar: 50 nm.

When preparing a MOF nanocarrier for drug delivery, several aspects have to be considered. These include the utilisation of non‐ or low toxic components and reactants, together with reaction conditions favouring the formation of nanosized crystals—having their optimum around or below 100 nm.^28^ Here we used ZIF‐8, which was prepared by mixing Zn^II^ ions and 2‐methylimidazole in water. The synthesis was carried out at room temperature. Optimal reaction conditions were found to be 5 min of reaction time to obtain a crystalline material of particle sizes around 68±15 nm as determined by transmission electron microscopy (TEM, Figure 1 and FigureS1, Supporting Information) by evaluating 100 nanoparticles.

As a precursor of ATO, we used sodium (*meta*)arsenite (NaAsO_2_) due to its high solubility in water. The pH value of its aqueous solution (7.7 mm) was adjusted to pH 7 with 1 m HCl to generate As(OH)_3_. The ZIF‐8 nanoparticles were kept in the solution at room temperature for 18 h. After that, ethanol was added to the reaction mixture to induce the particle precipitation. Subsequently, the drug loaded nanoparticles (denoted as As@ZIF‐8) were collected by centrifugation, washed well with deionized water to remove any non‐bound arsenic residues from the pores and dried under ambient pressure at 130 °C. In order to quantify and qualify the As^III^ drug in the ZIF‐8 framework, the prepared As@ZIF‐8 nanoparticles were analysed by various methods including Fourier‐transformed infrared (FT‐IR) spectroscopy and Raman spectroscopy, energy dispersive X‐ray (EDX) spectroscopy, inductively coupled plasma‐ optical emission spectrometry (ICP‐OES), CHN‐elemental analysis, argon sorption measurements and X‐ray powder diffraction (XRPD) analysis.

**Figure 2:**
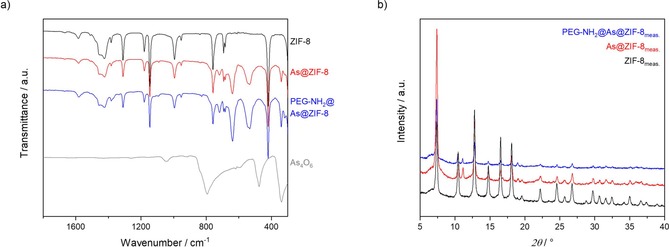
FT‐IR spectra in the area from 1800–300 cm^−1^ (a) and XRPD patterns (b) of ZIF‐8 (black), As@ZIF‐8 (red), PEG‐NH_2_@As@ZIF‐8 (blue) and As_4_O_6_ (grey).

ZIF‐8 is a neutral framework [Zn_1_[C_4_N_2_H_5_)_2_] and does not contain any free metal binding sites for arsenite anions. Therefore, to bind the anions to the framework, a ligand exchange has to take place. This hypothesis was supported by the results FT‐IR and Raman spectroscopy, elemental analysis and computational modelling. In the IR spectra we found new bands at 340, 540, 640, 720 and 1090 cm^−1^ (Figure 2 a, Figure S2 and S3, Supporting Information), which corresponded to the symmetric and asymmetric mode of As(OH)_2_ and As‐O stretching vibrations, and were in agreement with data reported in literature.^29^ Moreover, the results of Raman spectroscopy also supported this conclusion. New bands appeared at 320, 370, 600 and at 790 cm^−1^, and could be assigned to As‐OH bending, symmetric and antisymmetric stretching, and As‐O stretching vibrations (Figure S4 in the Supporting Information).^30^ These findings also ruled out the possibility that As(OH)_3_ was simply captured inside the pores and then dehydrated to As_4_O_6_ (Figure 2 a).^29^, ^31^ The expected loss of the imidazolate linker after the As‐loading was confirmed by CHN‐elemental analysis (Table S1). The virgin ZIF‐8 gave a sum formula of Zn_1_(C_4_N_2_H_5_)_1.95_ which corresponds to 0.05 missing linkers per zinc cation and can be considered as minor defects in the framework. However, after the loading of arsenite, the number of missing linkers per zinc atom clearly increases to 0.2 [Zn_1_(C_4_N_2_H_5_)_1.8_]. This suggests that some of the linkers of the framework were exchanged to arsenite anions. Based on this hypothesis, a structural model for As@ZIF‐8 was simulated (Figure S8). A modelled IR spectrum (Figure S9 and Table S2) matched well to the measured IR spectra and thus, supported our assumption of partial replacement of linker by arsenite. To quantify the As^III^ amount, we used two analytical methods—EDX spectroscopy and ICP‐OES analysis (Table 2). Whereas the EDX spectroscopy primarily gave information about the elemental composition of the surface levels, in ICP‐OES analysis the particles were decomposed in an aqueous solution of HNO_3_ prior to the measurement, and thus, the elemental compositions of the whole particle material were obtained. EDX spectroscopy revealed the Zn : As ratio of 1:0.33 (average of three measurements) which was in good agreement with the values found the by the ICP‐OES analysis which was 1:0.28 (average value of three measurements). These results clearly indicated that the As‐drug was loaded within the material pores and not just on the surface as it is often the case, especially, when non‐porous materials are used. The loaded amount of As corresponds to approximately 74 μg of As being loaded into 1 mg of carrier material (equivalent to 98 μg of As_2_O_3_/ 1 mg). This features a high loading capacity with respect to the fact that we used ZIF‐8−a non‐functionalized framework which intrinsically does not provide any accessible coordination sites. The drug loading is also clearly reflected in the results of the sorption analysis, since the specific surface area decreased from 1500 m^2^ g^−1^ to only 1150 m^2^ g^−1^ (Table 1, Figure S5, in the Supporting Information). Furthermore, the introduction of arsenite influenced the symmetry of the crystal structure as shown by the XRPD measurement (Figure 2 b). Variable temperature X‐ray powder diffraction and thermogravimetric analysis indicate that As@ZIF‐8 is stable up to 450 °C, which is slightly lower in comparison to the ZIF‐8 nanoparticles (Figure S6 and Figure S7). This is in agreement with our findings that some of the linkers were replaced by arsenite anions. In order to increase the biocompatibility and stability of the nanocarrier, the nanoparticle surface was coated with polyethylene glycol (PEG)—a polymer often used for the mentioned purposes.^32^ Here we used an amino‐functionalized derivative of polyethylene glycol (PEG‐NH_2_). In contrast to non‐functionalized PEG, coordinate interactions between the amino end‐groups of the polymer and zinc centres can take place, so that the polymer is bound to the surface and does not penetrate the pores of the framework.^33^ The successful coating was confirmed by FT‐IR spectroscopy (Figure 2 a and Figure S3, in the Supporting Information): new bands at 2870, 1250, 1100 and 840 cm^−1^ correspond to C−H bending vibrations, O−H and C−*O*−H stretching vibrations. Moreover, thermogravimetric analysis verified that 5.1 wt % PEG‐NH_2_ coating were present (Figure S7). ICP‐OES analysis of the coated samples (denoted as PEG‐NH_2_@As@ZIF‐8) revealed that the arsenic content was not affected by the coating (Table 2).

**Table 2:** Results of EDX spectroscopy, ICP‐OES and adsorption analysis of ZIF‐8, As@ZIF‐8 and PEG‐NH2@As@ZIF‐8.

Analysis	ZIF‐8	As@ZIF‐8	PEG‐NH_2_@As@ZIF‐8
EDX, *n*(Zn) : *n*(As)	1:0	1:0.33	1:0.39
ICP‐OES, *n*(Zn) : *n*(As)	1:0	1:0.28	1:0.30
Meas. spec. surface area (Ar, 77 K, m^2^ g^−1^)	1500	1150	490

### Drug release

**Figure 3:**
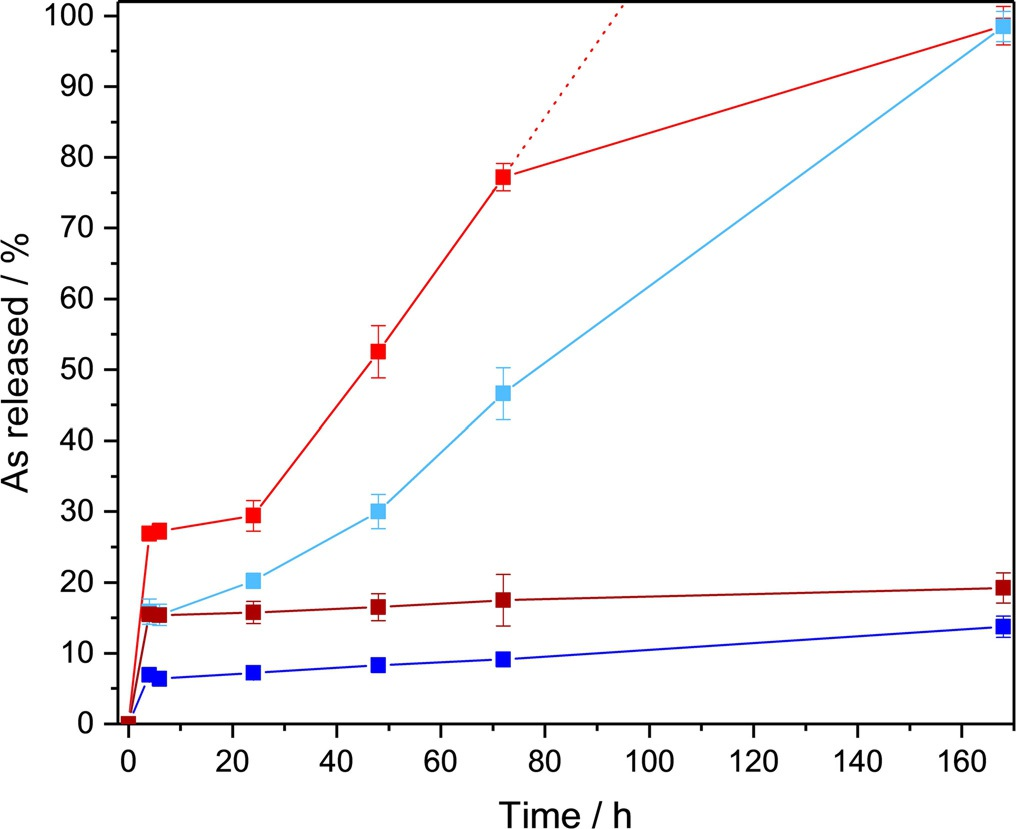
Arsenic‐release from As@ZIF‐8 and PEG‐NH_2_@As@ZIF‐8 at pH 6 (red, light blue, respectively) and pH 7.4 (dark red, dark blue, respectively) in a phosphate buffered saline at 37 °C, determined by ICP‐OES.

To enable a fundamental insight to the arsenic‐release kinetics of As@ZIF‐8 and PEG‐NH_2_‐coated As@ZIF‐8 in human physiological conditions, a simplified set‐up was utilized. Herein, we used a phosphate buffered saline of two different pH values (pH 6 and pH 7.4) at 37 °C, to mimic the microenvironment of tumorous‐ and healthy tissue.^27^ After 4, 6, 24, 48, 72 and 168 h the amount of arsenic and zinc released into the solution was determined by ICP‐OES (Figure 3 and Table S3 in the Supporting Information). At pH 7.4 (normal tissue environment) only 15.5 % arsenic was released after 24 h and a maximum of 19.2 % after 168 h. The solids were analysed by XRPD, FT‐IR and TG analyses. The results did not reveal any significant changes compared to the data of As@ZIF‐8 before the release studies, except for few new bands observed in IR spectra which could be assigned to phosphate absorbed from the buffered solution (Figure S10–S15). Additionally, no zinc could be detected in the solutions confirming that the carrier of ZIF‐8 did not decompose at pH 7.4. Interestingly, at pH 6 we detected a different behaviour compared to pH 7.4. At pH 6 (tumorous tissue environment) already 29.4 % of arsenic was released after 24 h and after 168 h all the amount of the originally loaded arsenic could be detected in the solution. The slope of the As‐release at pH 6 (Figure 3) suggests that the release was completed in less than 100 h. This fast arsenic release (compared to the release at pH 7.4) was caused by the framework decomposition of ZIF‐8 at pH 6 which was accompanied by a progressive formation of Na_6_Zn_6_(PO_4_)_6_⋅8 H_2_O from the released zinc ions and phosphate anions of the buffer. The framework decomposition and Na_6_Zn_6_(PO_4_)_6_ **⋅**8 H_2_O formation was verified by the XRPD, FT‐IR and TG analyses (Figure S16–21). The PEG‐NH_2_‐coated samples showed the same release trends as the corresponding non‐coated samples at pH 7.4 and pH 6. The drug release was only slightly slowed down due to the surface modification. At pH 7.4, the arsenic release dropped from 15.5 % to 7.2 % after 24 h, and the maximum release was 13.7 % instead of 19.2 % after 168 h. Also, at pH 6, the arsenic release declined from 29.4 % to 20.2 % in the first 24 h, and was completed after 168 h. This decelerated release behaviour of PEG‐NH_2_‐coated MOF nanoparticles has already been reported and was assigned to the decelerated particle decomposition and drug release due to a slower diffusion of the buffer solution though a layer of PEG.^34^, ^35^ Hence, the surface modification of the nanoparticles turned out to be a tool to slow down the drug release, especially in the first several hours. The arsenic release studies clearly showed that arsenic could be released much faster at slightly acidic conditions than at pH 7.4. This suggests that the material, when used as a nanocarrier, could keep the drug inside the pores at the conditions of normal tissues (and thus protect the environment from the drug effects), but rapidly release it at the more acidic microenvironment of cancer tissues.

### Cytotoxicity studies

In order to analyse the cytotoxic effect of As@ZIF‐8 and PEG‐NH_2_@As@ZIF‐8, an evaluation of the putative cytotoxic effect triggered by the different components of the nanoparticles, namely ZIF‐8, 2‐methylimidazole (the linker of ZIF‐8) and the original drug ATO is essential. For this purpose, we selected fibroblasts, as non‐tumorous cells and two ATRT cell lines—BT12 and BT16, which are known to respond positively to ATO treatment and incubated them with the nanoparticles and nanoparticle components for 24 h and 72 h.^20^ The amount of the administrated substances was calculated with respect to the fixed As‐ or Zn‐amount. First the composition of the nanoparticles of As@ZIF‐8 and PEG‐NH_2_@As@ZIF‐8 was determined by ICP‐OES (Table 2). Then the amount of the two samples (As@ZIF‐8 and PEG‐NH_2_@As@ZIF‐8) for the cytotoxicity studies was calculated with respect to a fixed concentration of arsenic corresponding to the concentration of As_2_O_3_ from 0.0001 to 100 μm. Corresponding to these amounts, the equivalent amount of ZIF‐8 and organic linker was calculated (for details, see Table S4 in the Supporting Information). After incubation time, cell viability was measured performing an MTT assay.^36^

**Figure 4:**
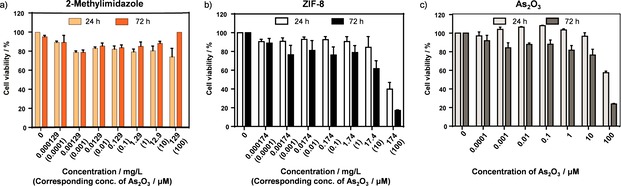
Cell viability of fibroblasts after 24 h and 72 h of incubation with different concentrations of (a) 2‐methylimidazole, (b) ZIF‐8 and (c) As_2_O_3_. The concentration given in mg L^−1^ corresponds to the amount of ZIF‐8 (or its linker needed to build it) which could be loaded with As_2_O_3_ in the concentration range of 0–100 μm (for details see Table S4 in the Supporting Information). Data are presented as mean ± S.E.M (n ≥3).

**Figure 5:**
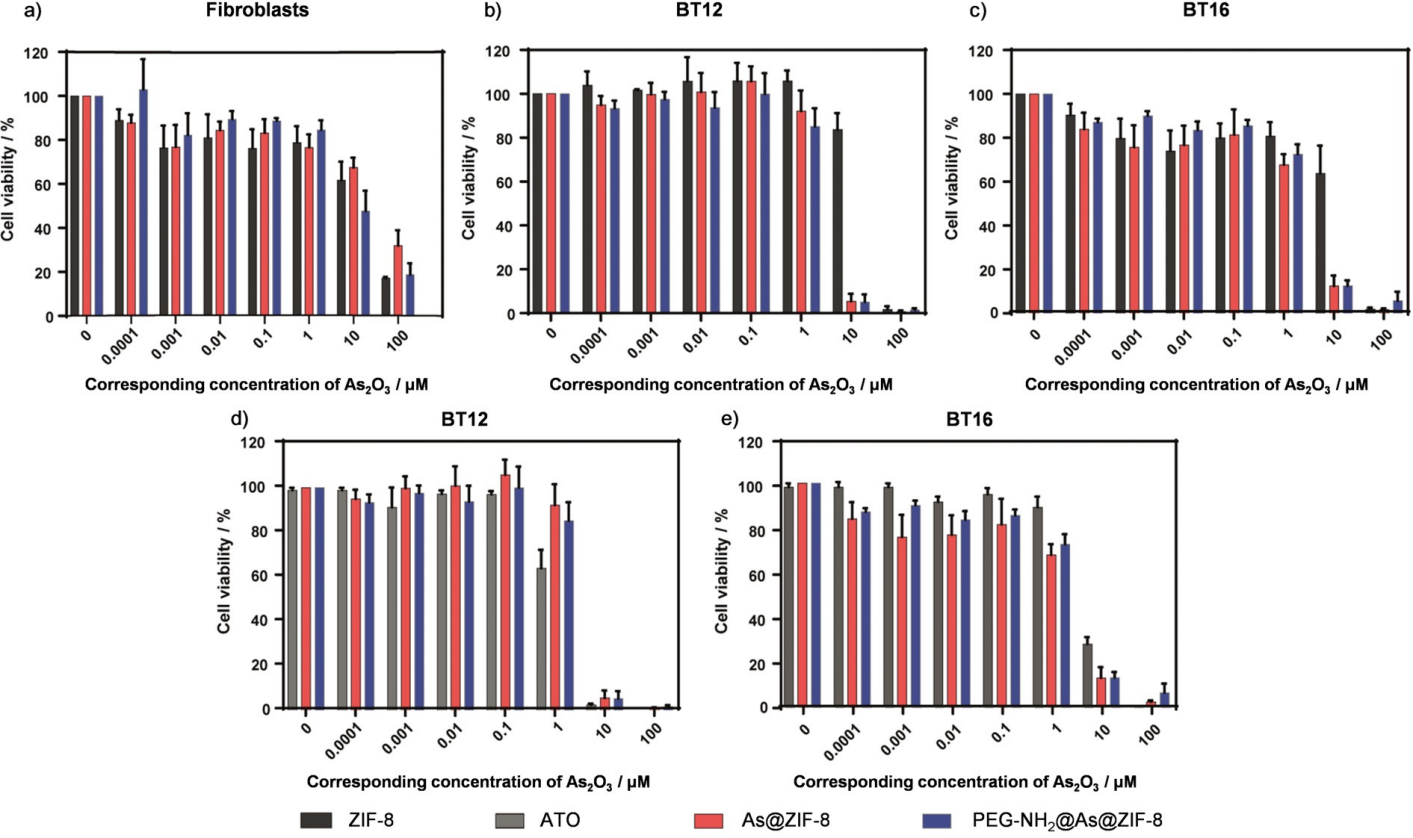
Cell viability of (a) fibroblasts, (b and d) BT12‐ and (c and e) BT16 cells after 72 h of incubation with different concentrations of ZIF‐8 (black), ATO (grey), As@ZIF‐8 (red) or PEG‐NH_2_@As@ZIF‐8 (blue). The given concentration corresponds to the concentration of As_2_O_3_ (0–100 μm) which was effectively loaded or could be theoretically loaded (for details see Table S4 in the Supporting Information). Data are presented as mean ± S.E.M (n≥3).

Analysis performed on fibroblasts showed that the organic linker only slightly decreased the cell viability after incubation (Figure 4 a) and therefore, its cytotoxic effect is negligible. In the next step we analysed the cell viability of fibroblasts treated with the nanocarrier itself. The results of ZIF‐8 did not show a significant cytotoxic effect after 24 h at concentrations lower than 174 mg L^−1^ (Figure 4 b). However, after 72 h the cell viability decreased for concentrations equal or higher than 17.4 mg L^−1^, suggesting a cytotoxic effect of ZIF‐8 at these high concentrations. Additionally, we analysed the cell viability of fibroblasts treated with arsenic‐loaded nanoparticles As@ZIF‐8 and PEG‐NH_2_@As@ZIF‐8 after 24 h and 72 h (Figure 5 a and Figure S22 in the Supporting Information). Interestingly, after 72 h the cytotoxic effect of both loaded nanoparticles was similar or lower than that of ZIF‐8 (Figure 5 a) indicating that the drug distributed via a MOF nanocarrier does not cause any additional cytotoxicity. The cytotoxicity of free arsenic trioxide against fibroblast was also tested (Figure 4 c) and corresponded to the IC_50_ value of 27.5 μm after 72 h.

To investigate the response of the ATRT cell lines BT12 and BT16 to the nanocarrier with and without the As‐drug cargo, we conducted the same experiments with ZIF‐8 and both arsenic‐loaded nanoparticles. We observed that there was a clear cytotoxic effect of the drug loaded nanoparticles compared to the empty ZIF‐8 nanocarrier already at an early time point and low drug concentration in BT12 and slightly later in BT16 (Figure S22 in the Supporting Information). After 72 h cell viability dramatically decreases in both cell lines for concentrations higher than 1 μm (Figure 5 b and c). This indicates that the specific effect of loaded arsenic drug is at least 5 times higher than the effect of ZIF‐8 alone (Table 3).

**Table 3:** Half‐maximal inhibitory concentrations (IC_50_ values; given as a corresponded concentration of As_2_O_3_; for details see Table S4 in the Supporting Information) for arsenic loaded MOFs and ZIF‐8 treatment of different ATRT cell lines, after 72 h.

	IC_50_ value [μm] after 72 h		
ATRT cell line	ZIF‐8	As@ZIF‐8	PEG‐NH_2_@As@ZIF‐8
BT12	12.5	2.8	2.4
BT16	12.8	1.7	2.1

Subsequent to these tests, we wanted to assess whether the specific cytotoxic effect of the arsenic drug contained in the MOFs was higher than an effect caused by free ATO. We compared the cytotoxicity driven by the two nanoparticle formulations (As@ZIF‐8 and PEG‐NH_2_@As@ZIF‐8) and ATO on BT12‐ and BT16 cell lines at 24 h (Figure S23, in the Supporting Information) and 72 h (Figure 5 d and e). In summary, the cytotoxic effect of free ATO and arsenic drug contained in MOFs was comparable. In the case of BT12 (Figure 5 d), ATO showed a higher cytotoxic effect at 1 μm and a comparable effect up to 10 μm. On the contrary, in BT16 cells (Figure 5 e), ATO presented a lower cytotoxic effect than the loaded nanoparticles at every analysed concentration. In conclusion, both nanoparticles, As@ZIF‐8 and PEG‐NH_2_@As@ZIF‐8, trigger specific cytotoxicity at low concentrations in rhabdoid tumour cell lines. The low therapeutic doses would avoid possible toxicity caused by ZIF‐8 in non‐tumorous cells, like fibroblasts, when applied in vivo.

## Conclusions

In this work we synthetized ZIF‐8 in a nanoparticle formulation and successfully loaded arsenite into its pores. The anionic As‐drug was introduced to the neutral framework via post‐synthetic ligand exchange. To enhance the material biocompatibility and gain a better control over the drug release, we modified the nanoparticle surface with amino‐functionalized polyethylene glycol. Both arsenic loaded nanoparticles, As@ZIF‐8 and coated As@ZIF‐8, showed a loading capacity as high as 74 μg of As per 1 mg of material. The arsenic release from the prepared nanocarriers was investigated in phosphate buffered saline at two different pH values (pH 7.4 and pH 6.0). The results showed that ZIF‐8 nanoparticles released only a little arsenic at the neutral pH, that is, a pH value of healthy tissues and blood, whilst a complete arsenic release took place at the more acidic pH value, that is, a pH value found in some tumours tissues. This was due to a nanocarrier decomposition at the more acidic pH value, which was perfectly justified by analysing the material which remained after the release tests. In vitro cytotoxicity studies showed that the individual fragments of the nanoparticle formulations, ZIF‐8 and 2‐methylimidazole did not cause critical harm to the fibroblast cell line at moderate concentrations. Additionally, the cytotoxic effect of both As‐drug loaded nanoparticles was similar or lower than that of ZIF‐8. On the contrary, both As‐loaded nanocarriers showed a substantial cytotoxic effect on cancer cell lines at low concentrations. Moreover, the As‐loaded nanoparticles presented a comparable cytotoxic effect to free arsenic trioxide.

All in all, the framework of ZIF‐8 fulfilled essential requirements for an arsenic drug delivery carrier for cancer therapy such as: a nanoparticle size, high arsenic loading, very prominent pH‐triggered release behaviour and promising results of the first in vitro experiments. Taking into account the low cytotoxicity of the drug loaded nanoparticles on fibroblast and their cytotoxicity on the selected cancer cell lines, which was comparable to the free drug, the presented material is a very promising candidate for drug delivery of arsenic trioxide. Such material is expected to exhibit the desired advantage of the safe drug delivery within the body and drug release triggered by a pH change in the vicinity of the tumour. As the next steps, we plan to carry out further in vitro and in vivo studies to evaluate the carrier performance with other cancer cell lines and small rodents.

## Experimental Section

### Materials and methods

All reagents were of analytical grade and used as received from commercial suppliers: zinc nitrate hexahydrate, sodium (*meta*)arsenite, 2‐methylimidazole and arsenic trioxide from Sigma–Aldrich, and alpha monomethoxy‐omega‐amino poly(ethylene glycol) from Iris Biotech GmbH. Fourier transform infrared (FT‐IR) spectra were recorded in the range of 180–4000 cm^−1^ or 400–4000 cm^−1^ on a Bruker Equinox 55 FT‐IR spectrometer equipped with an ATR unit. Raman spectra were recorded with a Thermo Scientific DXR Raman‐Microscope in the range 1800‐35 cm^−1^ using a 532 nm laser operated with 10 mW power. The samples were illuminated for 300 s (20‐fold magnification, 50 μm slit aperture, high resolution grating (1800 lines mm^−1^), spectral resolution 1 cm^−1^). Thermogravimetric analysis (TGA) was measured on a TA Instruments Q500 device in a temperature range of 25–700 °C under a nitrogen atmosphere at a heating grade of 10 K min^−1^. X‐ray powder diffraction data were collected in the 4–40° 2θ range using a Seifert XRD 3003 TT—powder diffractometer with a Meteor1D detector operating at room temperature using Cu K_α1_ radiation (*λ*=1.54187) as well as with a Bruker D8 Advance diffractometer with Cu‐*K*_α_ radiation (*λ*=1.54184) with a 1D LynxEye detector, for that the samples were ground and filled into a Hilgenberg glass capillary (outer diameter 0.3 mm, wall thickness 0.01 mm). For variable temperature X‐ray powder diffraction (VTXRPD) the samples were ground, filled into a silica‐glass Hilgenberg glass capillary (outer diameter 0.3 mm, wall thickness 0.01 mm) and measured between *T*=50 and 650 °C with steps of 0.02° and an acquisition time 3 s per step and transmission geometry. TEM images were recorded a JEM 2100F microscope (JEOL) with a FEG electron source operated at 20 kV. Samples were prepared by depositing a drop of the crystalline products dispersed in ethanol onto carbon‐coated copper grids (200 mesh) and dried in air. The size of the nanoparticles was determined from calibrated TEM images using ImageJ software.^37^ One hundred particles were analysed to determine the average size. The elemental composition of solid samples was determined by energy dispersive X‐ray spectroscopy (EDX) with the Philips XL 30 FEG with a EDAX SiLi detector, while liquid samples were analysed by ICP‐OES with the Vista MPX of VARIAN with arsenic and zinc standard solution of 10 ppm and 20 ppm. CHN‐elemental analysis was measured with a Vario EL III (Elementar‐Analysensysteme GmbH). Argon‐gas sorption isotherms were measured with a Quantachrome Autosorb‐I ASI‐CP‐ 8 instrument. Argon‐sorption experiments were performed at 77.3 K in the range of 5.00×10^−5^ ≤ *P*/*P*_0_ ≤ 1.00 with Ar. Cells were cultivated in a Heracell™ 150i CO_2_ incubator (Thermo Scientific). MTT assay was analysed using a Multiskan Ascent Mircoplate Reader (Thermo Electron Corporation).

### Synthesis of ZIF‐8 [Zn(C_4_H_5_N_2_)_2_]_*n*_ nanoparticles^38^

Nanoparticles or ZIF‐8 were prepared according the reported procedure as follows.^38^ Zinc nitrate hexahydrate (3.9 mmol, 1.17 g) and 2‐methylimidazole (0.28 mol, 22.7 g) were dissolved in 8 mL and 80 mL deionized water, respectively. The two solutions were mixed and kept at room temperature for 5 minutes. After that, the product was collected via centrifuge with 5000 rpm for 30 minutes, washed with deionized water twice (each time 50 mL), and finally dried at 130 °C and ambient pressure for 2 h to obtain 450 mg of ZIF‐8 nanoparticles. The material was characterized by XRPD, FT‐IR, Raman, TGA, TEM, CHN‐elemental analysis and sorption analysis.

### Drug loading

An aqueous sodium (*meta*)arsenite solution (7.7 mm) with pH 7 was prepared by dissolving sodium (*meta*)arsenite NaAsO_2_ (1 g, 7.7 mmol) in 1 L of distilled water and subsequent adjustment of the pH with 1 m hydrochloric acid. 500 mg prepared ZIF‐8 nanoparticle were dispersed in 250 mL of the freshly prepared 7.7 mm aqueous arsenite solution and kept at room temperature for 18 h, collected via centrifuge with 5000 rpm for 30 minutes, washed with deionized water (2x 50 mL) and finally dried at 130 °C and ambient pressure for 2 h. The drug loaded material [As@ZIF‐8] was characterised by XRPD, FT‐IR, Raman, TGA, TEM, EDX, ICP‐OES, CHN‐elemental and sorption analysis.

Coating drug loaded nanoparticles with amino‐functionalized polyethylene glycol

250 mg of the loaded ZIF‐8 nanoparticles were dispersed in 25 mL of a 1.6 mm aqueous solution of alpha monomethoxy‐omega‐amino poly(ethylene glycol) (PEG‐NH_2_) and kept at room temperature. After 2 h the sample [PEG‐NH_2_@As@ZIF‐8] was collected by centrifuge and washed with deionized water (3×20 mL) and dried under ambient pressure at 130 °C. The material was characterised by XRPD, FT‐IR, Raman, TGA, TEM, EDX, ICP‐OES and sorption analyses.

### Drug release

10 mg of As@ZIF‐8 or PEG‐NH_2_@As@ZIF‐8 were dispersed in 10 mL of 0.01 m phosphate buffer at pH 7.4 or at pH 6. At a certain period of time (after 4, 6, 24, 48, 72 or 168 h), 1 mL of the solution was removed for analysis and replaced by 1 mL of a fresh phosphate buffer solution. The amount of arsenic and zinc in the taken 1 mL sample was determined by ICP‐OES analysis. The arsenic‐release studies were done in triplicates, data are presented as mean ± standard deviation. Moreover, the remained nanoparticles were analysed at each time step by XRPD, FT‐IR, TGA, and EDX analyses.

### Cell culture

ATRT cell lines were cultured in suspension with DMEM/F12‐Medium supplemented with B27 supplement (2 %), N2 supplement (1 %), penicillin and streptomycin (1 %), EGF (20 ng mL^−1^) and FGF 20 ng mL^−1^. BT12 cells were a gift from Dr. Marc Remke (University of Düsseldorf, Germany) and BT16 cells were received as a gift from Dr. Martin Hasselblatt (University of Muenster, Germany). Human fibroblasts of the respiratory system of healthy donors were provided from Niki Loges (University of Muenster, Germany). All cells were cultured in 5 % CO_2_ at 37 °C. The identity of all cell lines was confirmed by STR‐PCR.

### Cytotoxicity studies

Cells were seeded in a 96‐well plate at a density of 4000 (fibroblasts), 4.000 (BT16) or 8.000 (BT12) cells per well. After 24 h, cells were treated with increasing concentrations of ZIF‐8, As@ZIF‐8, PEG‐NH_2_@As@ZIF‐8 or ATO and incubated for 24 h or 72 h. The amount of the samples was calculated with regard to the fixed amount of As and Zn (corresponding to the concentration range of As_2_O_3_ from 0 to 100 μm which was effectively loaded or could be theoretically loaded; for details, see Table S4, in the Supporting Information). On the day of measurement, 10 μL of MTT reagent was added. Viable cells convert tetrazolium dye MTT (3‐(4,5‐dimethylthiazol‐2‐yl)‐2,5‐diphenyltetrazolium bromide) into an insoluble, purple‐coloured formazan dye. After 3 h incubation time, formazan crystals were resuspended with 100 μL isopropanol ‐HCl (0.04 N). MTT assay^36^ was performed using a Multiskan Ascent Mircoplate Reader (Thermo Electron Corporation). The absorbance was measured at a wavelength of 570 nm and a reference wavelength of 630 nm. Data was analysed using GraphPad Prism software version 7.00.

## Conflict of interest

The authors declare no conflict of interest.

## Supporting information

As a service to our authors and readers, this journal provides supporting information supplied by the authors. Such materials are peer reviewed and may be re‐organized for online delivery, but are not copy‐edited or typeset. Technical support issues arising from supporting information (other than missing files) should be addressed to the authors.

Supplementary
