# Supplementary material for: Zeolitic Imidazolate Framework‐8 as pH‐Sensitive Nanocarrier for “Arsenic Trioxide” Drug Delivery
Source: Chemistry. 2019 Sep 13;25(57):13189–96. doi: 10.1002/chem.201902599 (PMC6856809; doi:10.1002/chem.201902599)
Supplement: Supplementary file 1 — Supplementary [file CHEM-25-13189-s001.pdf]

CHEMISTRY
A European Journal

Supporting Information

Zeolitic Imidazolate Framework-8 as pH-Sensitive Nanocarrier for "Arsenic Trioxide" Drug Delivery

Romy Ettlinger+,[a] Natalia Moreno+,[b] Dirk Volkmer,[a] Kornelius Kerl,[b] and Hana Bunzen*[a]

chem_201902599_sm_miscellaneous_information.pdf

Electronic Supplementary Information (ESI) for:

Zeolitic Imidazolate Framework-8 as pH-sensitive Nanocarrier for 'Arsenic Trioxide' Drug Delivery

Romy Ettlinger,[a] Natalia Moreno,[b] Dirk Volkmer,[a] Kornelius Kerl [b] and Hana Bunzen*[a]

[a] Chair of Solid State and Materials Chemistry, Institute of Physics, University of Augsburg, Universitätsstraße 1, D-86159 Augsburg, Germany
[b] University Children´s Hospital Muenster, Pediatric Hematology and Oncology, Albert-Schweitzer-Campus 1, D-48149 Muenster

Contents

Characterization of ZIF-8, As@ZIF-8 and PEG-NH_2@As@ZIF-8 S2
Computational modelling of As@ZIF-8 S6
Drug release and MOF stability studies S8
Cytotoxicity studies S15
References S17

Characterization of ZIF-8, As@ZIF-8 and PEG-NH_2@As@ZIF-8

Transmission electron microscopy (TEM)

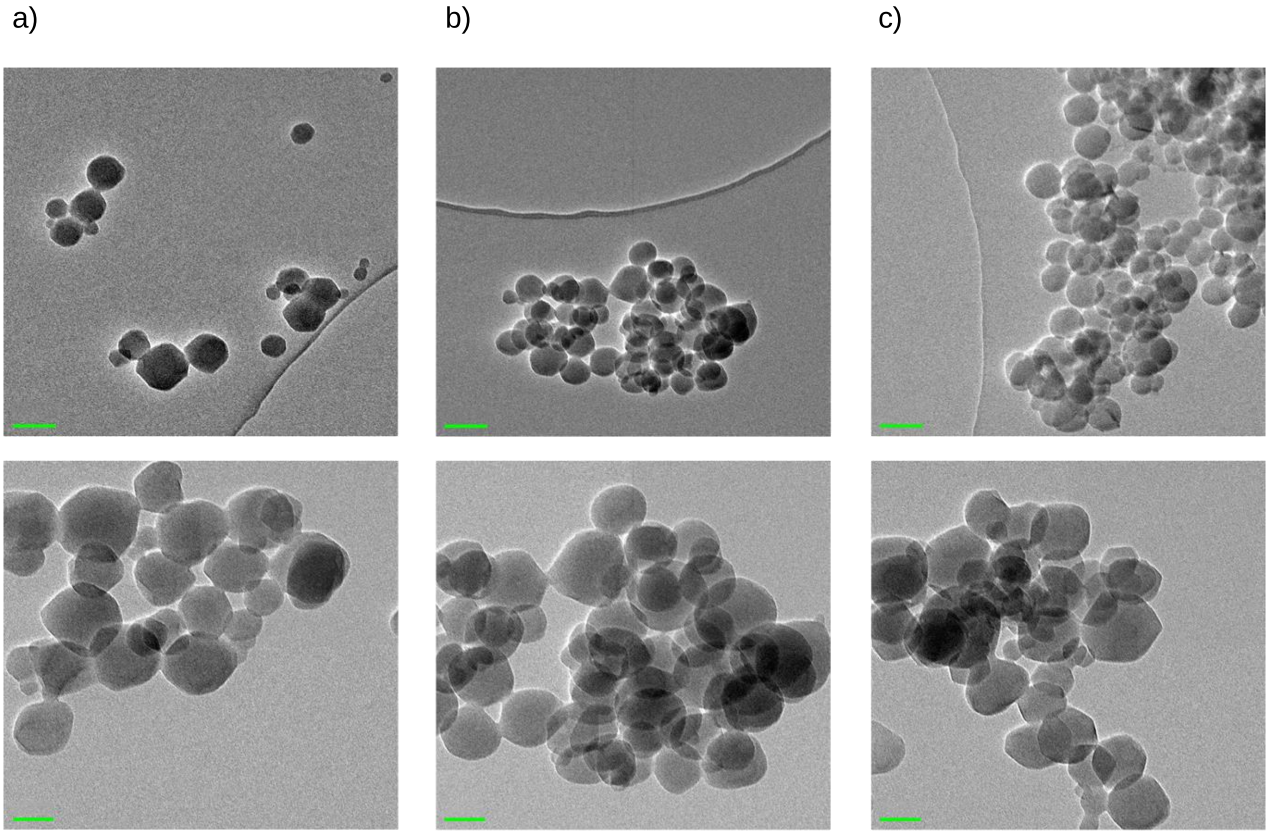

Figure S1. TEM micrographs of ZIF-8 nanoparticles before (a) and after (b) the drug loading, and after the coating with PEG-NH_2 (c); scale bar: 100 nm (top line), 50 nm (bottom line).

FTIR spectroscopy

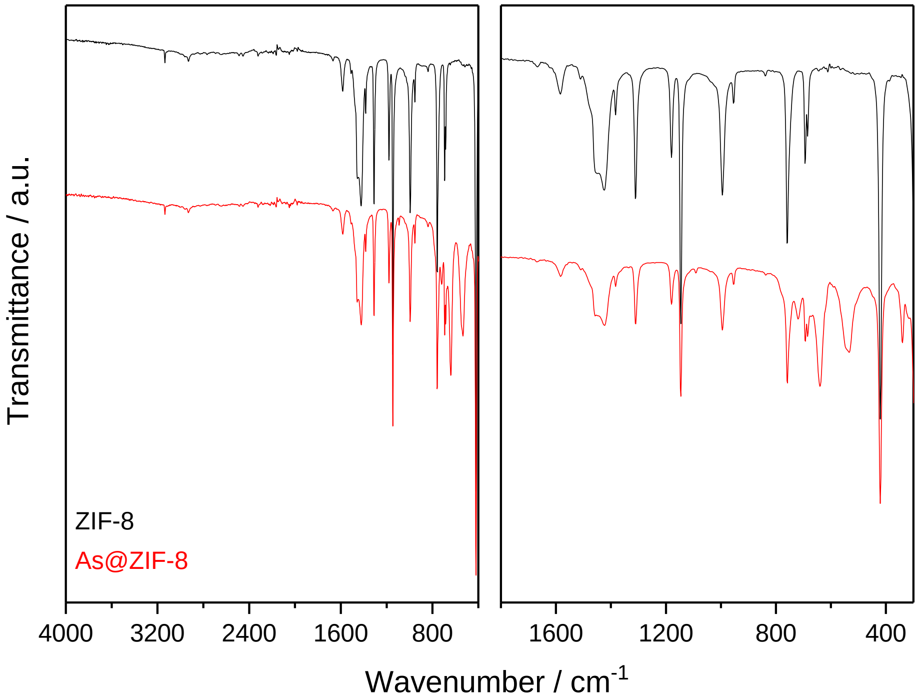

Figure S2. Comparison of IR spectra of ZIF-8 (black) and As@ZIF-8 (red) the area from 4000 to 400 $cm^{-1}$and from 1800 to 300 cm^{-1}.

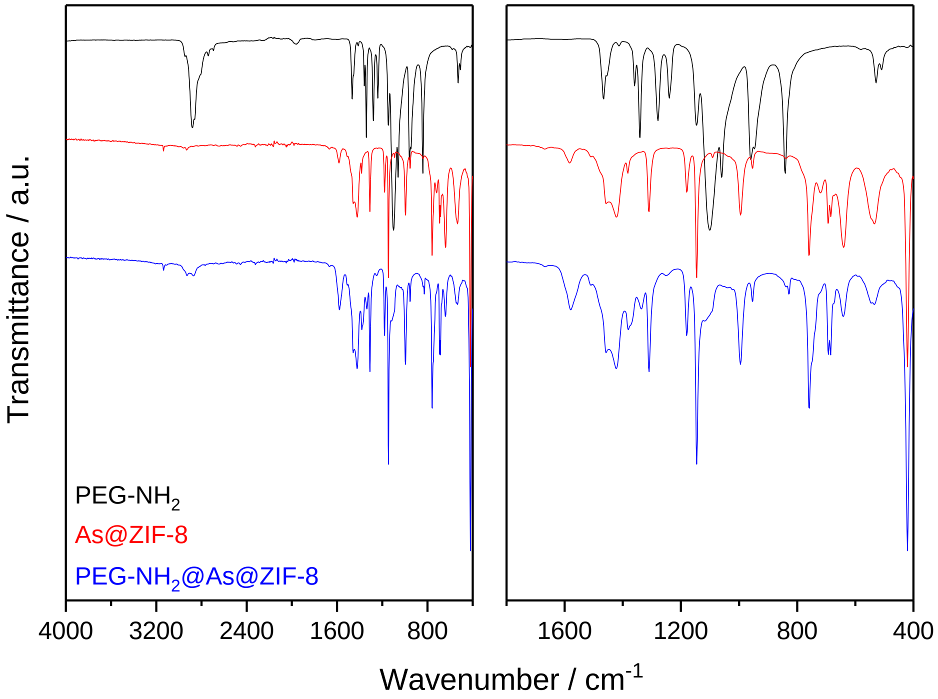

Figure S3. Comparison of IR spectra of PEG-NH_2 (black), As@ZIF-8 (red) and PEG-NH_2@As@ZIF-8 (red) in the area from 4000 to 400 cm^{-1} and 1800 to 400 cm^{-1}.

Raman spectroscopy

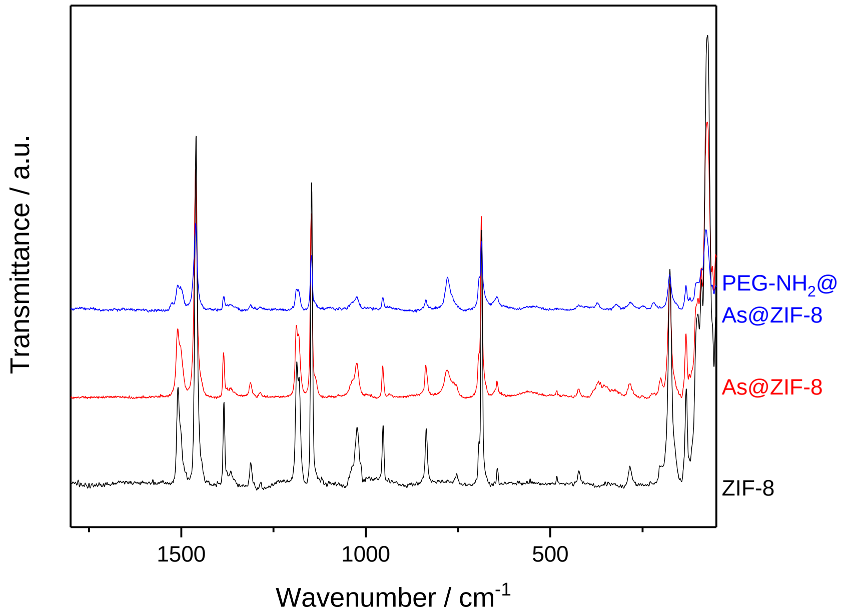

Figure S4. Comparison of Raman spectra of ZIF-8 (black), As@ZIF-8 (red) and PEG-NH@ As@ZIF-8 (blue) in the area from 1800 to 50 cm^{-1}.

Sorption analysis

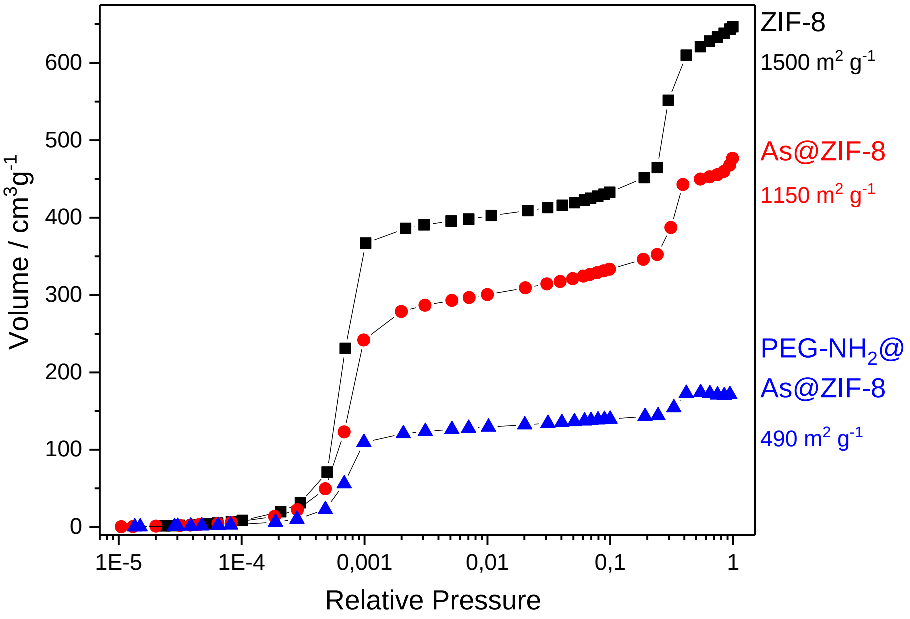

Figure S5. Argon adsorption isotherms for ZIF-8 (black), As@ZIF-8 (red) and PEG-NH_2@As@ZIF-8 (blue) at 77 K.

Variable temperature X-ray powder diffraction (VT XRPD)

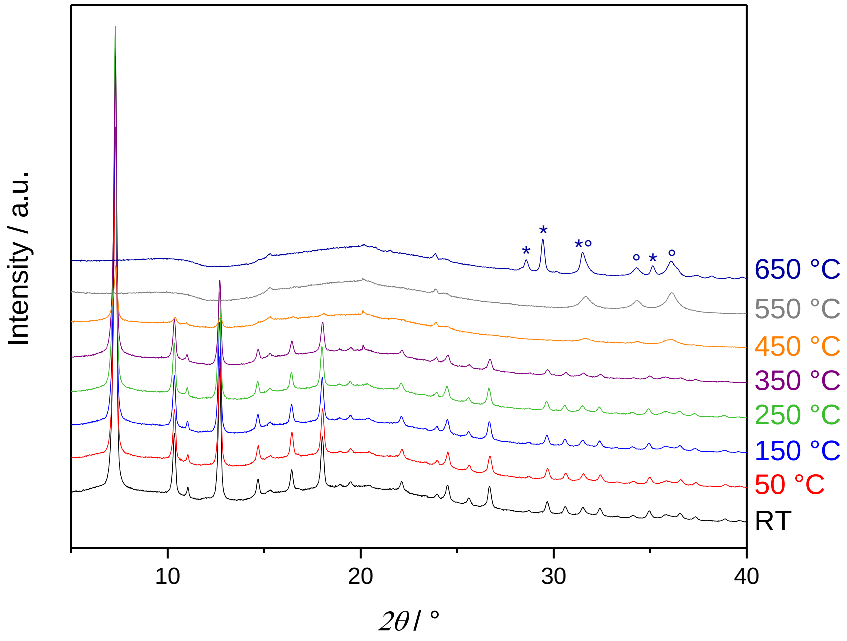

Figure S6. VT XRPD patterns of As@ZIF-8 in the range of RT-650 °C, decomposing to ZnO (**°**, ICSD: 57450) and $Zn_4(AsO_4)_2O$ (*****, ICSD: 404034).

Thermogravimetric analysis

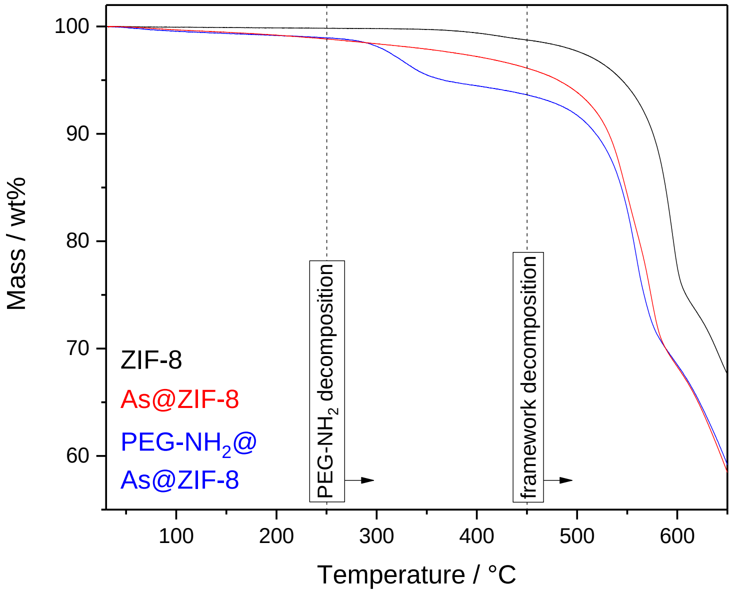

Figure S7. Thermogravimetric analysis of activated ZIF-8 (black), As@ZIF-8 (red) and PEG-NH_2@As@ZIF-8 (sample exposed to a flow of nitrogen gas).

CHN-elemental analysis

Table S1. Elemental composition of ZIF-8 and As@ZIF-8 determined by CHN-elemental analysis, analysis was done in triplicates, data are presented as mean ± standard deviation.

	ZIF-8 (wt%)	As@ZIF-8 (wt%)
N	26.56 ± 2.26	20.01 ± 0.19
C	42.07 ± 0.10	34.77 ± 0.32
H	2.13 ± 0.76	0.78 ± 0.08

Computational modelling

In order to simulate the crystal structure and vibrational properties of As@ZIF-8, first principle DFT+D lattice geometry optimizations were performed with the CASTEP code,[1] PBE-GGA, on-the-fly generated norm-conserving plane-wave pseudopotentials (energy cutoff: 1380 eV) employing a semi-empirical dispersion correction for non-bonding interactions according to the scheme suggested by Tkatchenko and Scheffler.[2,3]
The final lattice parameters and selected properties of the fully converged cell showing the best match with the experimental vibrational frequencies is shown in **Table S2**. A lattice model of the geometry-converged unit cell of this compound is displayed in **Figure S8**. Phonon frequencies were obtained via CASTEP DFPT linear response calculations at the Γ points of the converged primitive cell geometries.[4] Selected calculated frequencies are presented in **Table S2** together with an assignment to characteristic (localized = vibrational) modes. The calculated and measured IR spectra were compared and fitted with SpecDis version 1.71 software.[5] Herein, a scaling factor of 1.003 was applied and the intensity of the calculated spectra was divided by a factor of 65 and 20 in the range of 1800-200 cm^{-1} and 800-200 cm^{-1}, respectively.

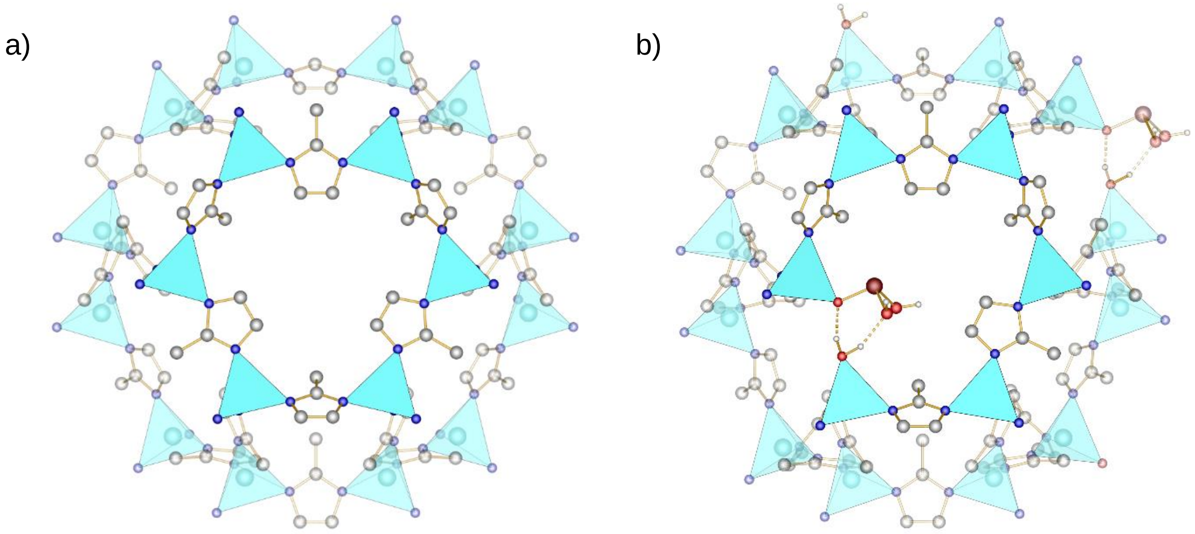

Figure S8. Comparison of the polyhedra packing model of the crystal lattices of (a) ZIF-8 and (b) As@ZIF-8. (C: grey; H: white; O: red; N: blue; Zn-tetrahedral: light blue; As: dark red).

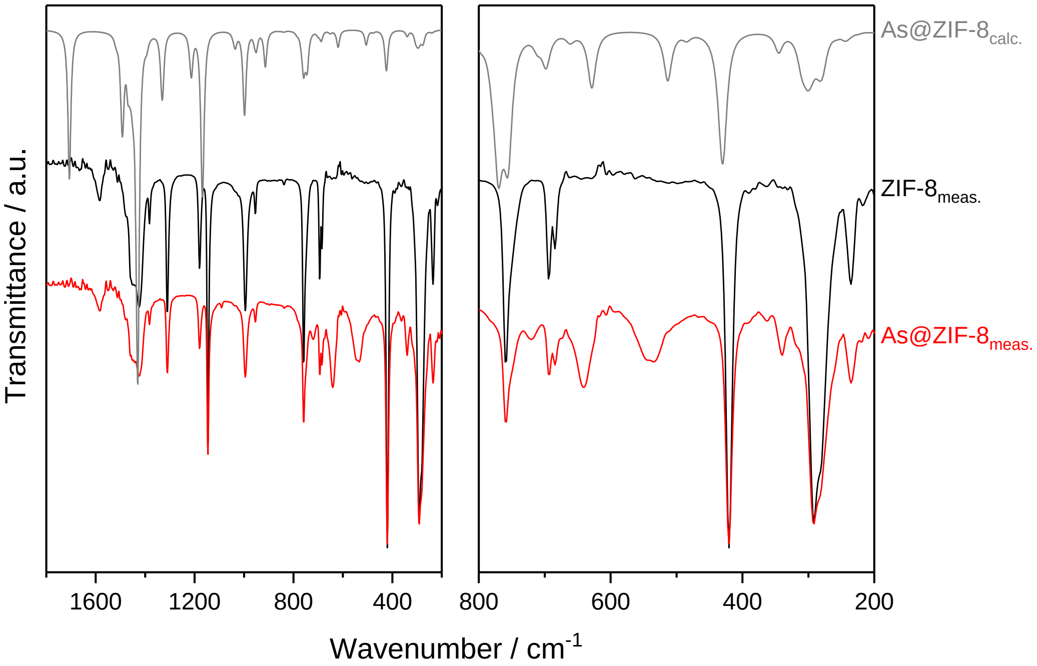

Figure S9. Comparison of measured FTIR spectra of ZIF-8 (black) and As@ZIF-8 (red) with modelled IR spectra of As@ZIF-8 (grey) in the area from 1800 to 200 cm^{-1} (left) and 800 to 200 cm^{-1} (right).

Table S2. Additional vibrational frequencies observed in the modelled IR spectrum of As@ZIF-8 in comparison to the modelled IR spectrum of ZIF-8 (both shown in Fig. S9).

Vibrational frequency / cm^{-1}	Vibrational mode
270 (w) , 300 (w) , 333 (m)	H-O-As angular deformation
414 (m)	(HO)-As-O angular deformation
495 (s), 607 (s)	As-O-H ⋯ (H-O-H) combination tone
729 (s)	(Zn-O-As) ⋯ (Zn-O-H) combination tone
895 (s) , 931 (m)	(As-O-H) ⋯ (Zn-O-H) combination tone

Drug release and MOF stability studies

Drug release followed over 7 days

Table S3. Amount of arsenic (%) released from As@ZIF-8 and PEG-NH_2@As@ZIF-8 into a phosphate buffer solution at 37 °C at pH 6 and pH 7.4, determined by ICP-OES.

Time	pH 6	pH 6 (PEG-NH_2 coated)	pH 7.4	pH 7.4 (PEG-NH_2 coated)
4 h	26.9 ± 1.0	15.9 ± 1.8	15.5 ± 0.8	6.9 ± 1.0
6 h	27.2 ± 1.0	15.4 ± 1.5	15.4 ± 0.9	6.4 ± 1.0
24 h	29.4 ± 2.1	20.2 ± 1.1	15.8 ± 1.5	7.2 ± 1.0
48 h	52.5 ± 3.6	30.0 ± 2.4	16.5 ± 1.9	8.3 ± 1.0
72 h	77.2 ± 1.9	46.6 ± 3.7	17.5 ± 3.6	9.2 ± 1.1
168 h	98.6 ± 2.7	98.5 ± 2.1	19.2 ± 2.1	13.7 ± 1.5

MOF stability in phosphate buffered saline at pH 7.4 followed over 7 days

X-ray powder diffraction (XRPD) measurements:

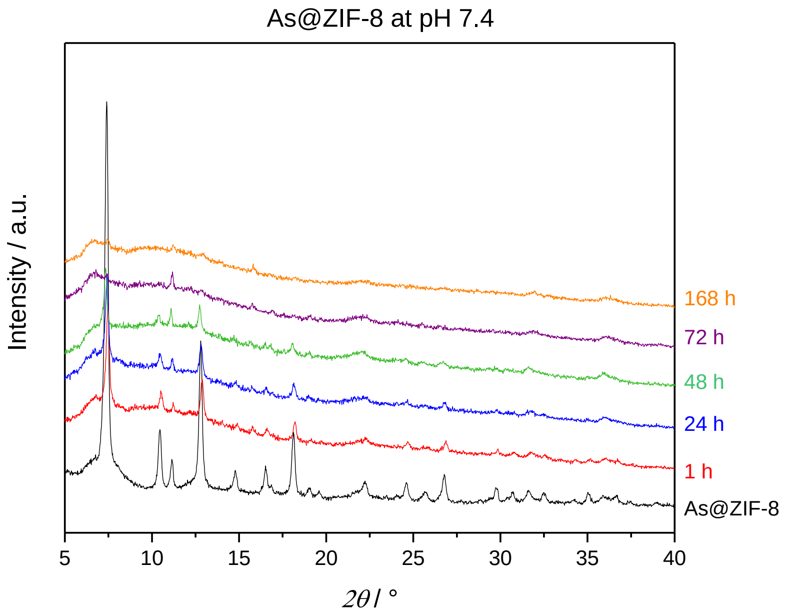

Figure S10. Comparison of measured XRPD patterns of As@ZIF-8 (black), As@ZIF-8 after the arsenic release studies carried out at pH 7.4 for 1 h (red), 24 h (blue), 48 h (green), 72 h (purple) and 168 h (orange).

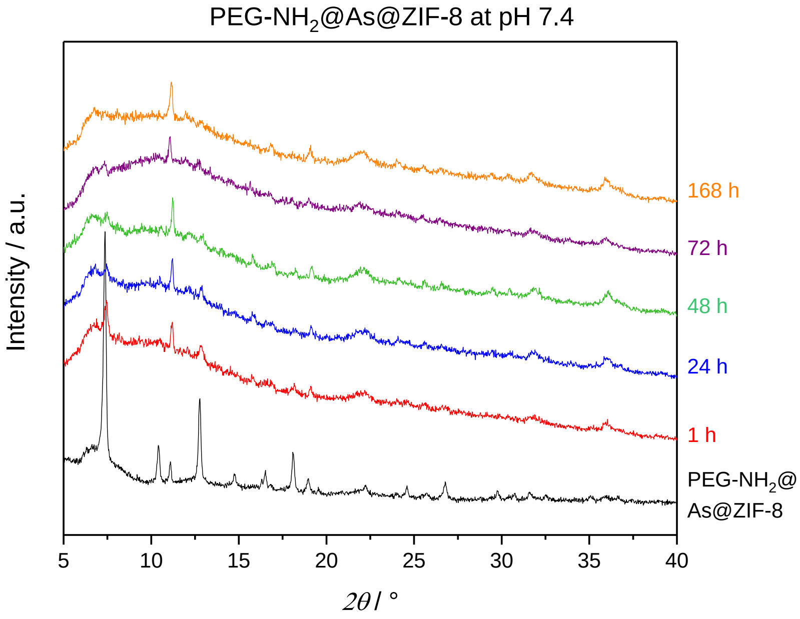

Figure S11. Comparison of measured XRPD patterns of PEG-NH_2@As@ZIF-8 (black), PEG-NH_2@As@ZIF-8 after the arsenic release studies carried out at pH 7.4 for 1 h (red), 24 h (blue), 48 h (green), 72 h (purple) and 168 h (orange).

FTIR spectroscopy:

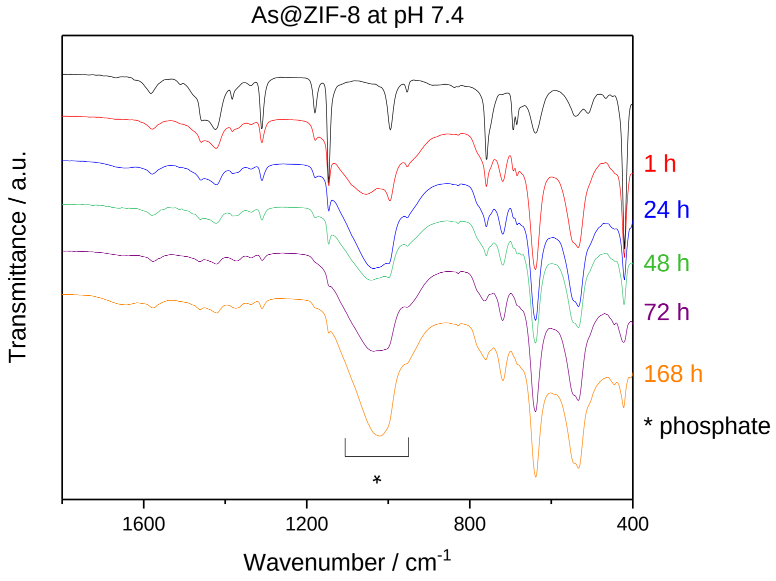

Figure S12. FTIR spectra of As@ZIF-8 (black) and As@ZIF-8 after the arsenic release studies carried out at pH 7.4 for 1 h (red), 24 h (blue), 48 h (green), 72 h (purple) and 168 h (orange) displayed in the area from 1800 to 400 cm^{-1}.

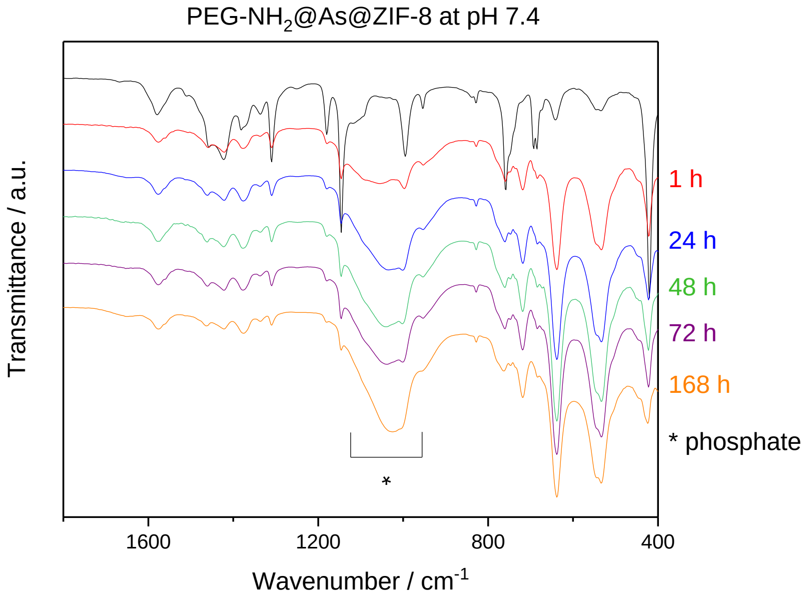

Figure S13. FTIR spectra of PEG-NH_2@As@ZIF-8 (black) and PEG-NH_2@As@ZIF-8 after the arsenic release studies carried out at pH 6 for 1 h (red), 24 h (blue), 48 h (green), 72 h (purple) and 168 h (orange) displayed in the area from 1800 to 400 cm^{-1}.

Thermogravimetric analysis:

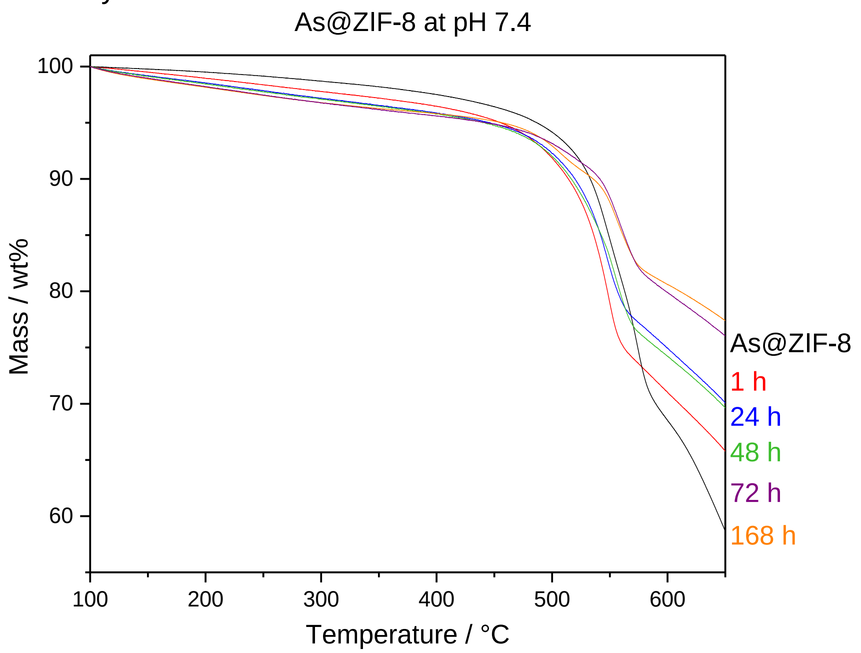

Figure S14. Thermogravimetric analysis of As@ZIF-8 (black) before and after the arsenic release studies carried out at pH 7.4 for 1 h (red), 24 h (blue), 24 h (green), 72 h (purple) and 168 h (orange) (samples were activated at 100 °C for 3 h and exposed to a flow of nitrogen gas).

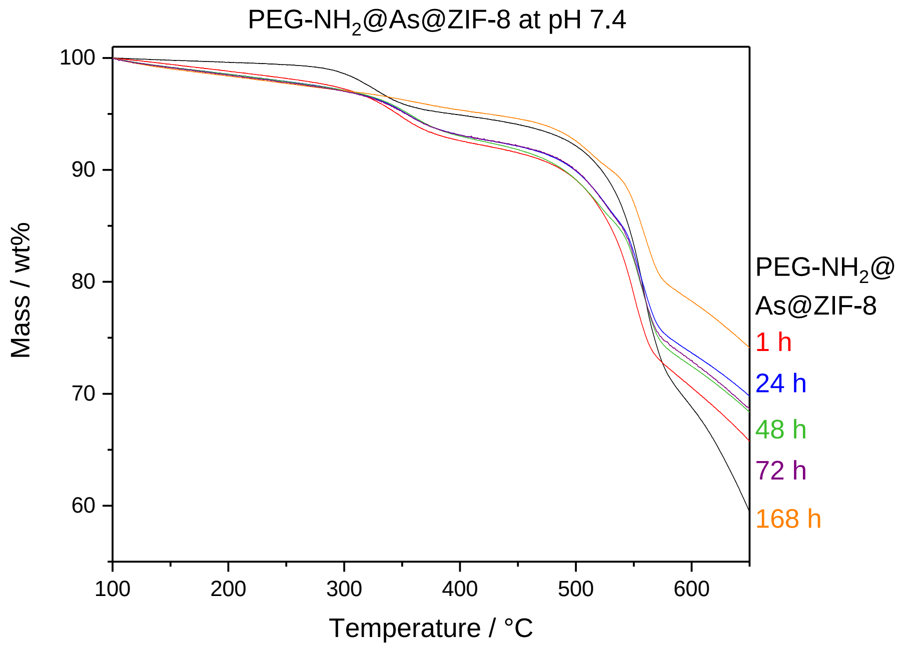

Figure S15. Thermogravimetric analysis of PEG-NH_2@As@ZIF-8 (black) before and after the arsenic release studies carried out at pH 7.4 for 1 h (red), 24 h (blue), 24 h (green), 72 h (purple) and 168 h (orange) (samples were activated at 100 °C for 3 h and exposed to a flow of nitrogen gas).

MOF stability in phosphate buffered saline at pH 6 followed over 7 days

X-ray powder diffraction (XRPD) measurements:

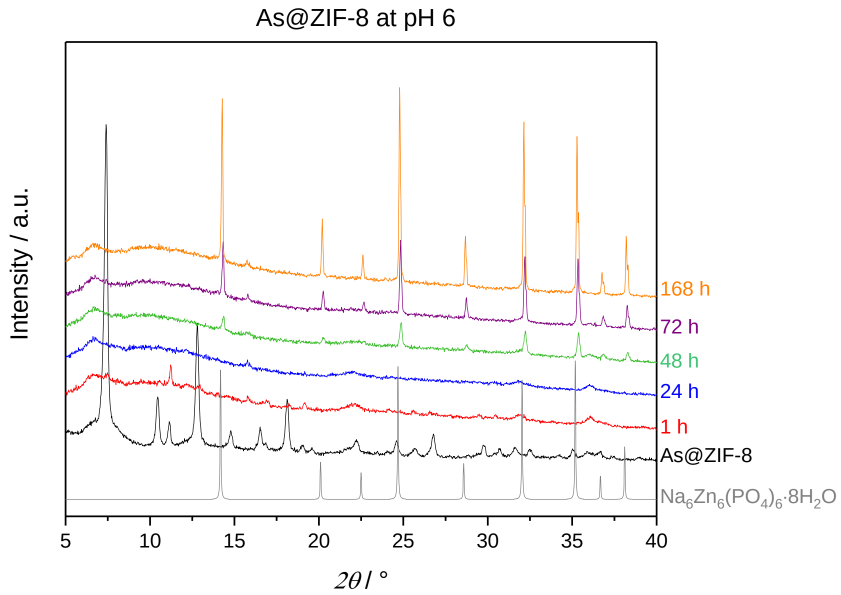

Figure S16. Comparison of measured XRPD patterns of As@ZIF-8 (black), As@ZIF-8 after the arsenic release studies carried out at pH 6 for 1 h (red), 24 h (blue), 48 h (green), 72 h (purple) and 168 h (orange) and the calculated XRPD pattern of $Na_6Zn_6(PO_4)_6 \cdot 8H_2O$ (grey, CSD: 56499).

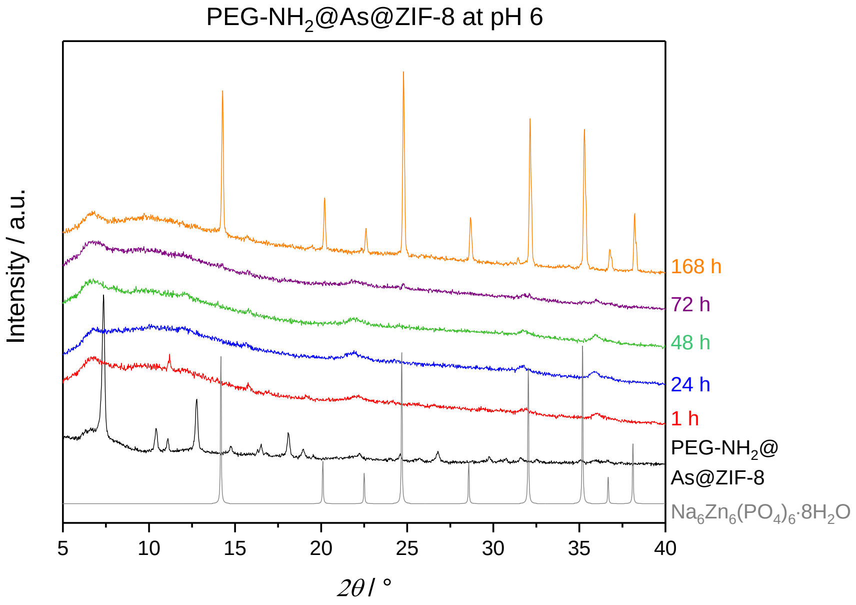

Figure S17. Comparison of measured XRPD patterns of PEG-NH_2@As@ZIF-8 (black), PEG-NH_2@As@ZIF-8 after the arsenic release studies carried out at pH 6 for 1 h (red), 24 h (blue), 48 h (green), 72 h (purple) and 168 h (orange) and the calculated XRPD pattern of $Na_6Zn_6(PO_4)_6 \cdot 8H_2O$ (grey, CSD: 56499).

FTIR spectroscopy:

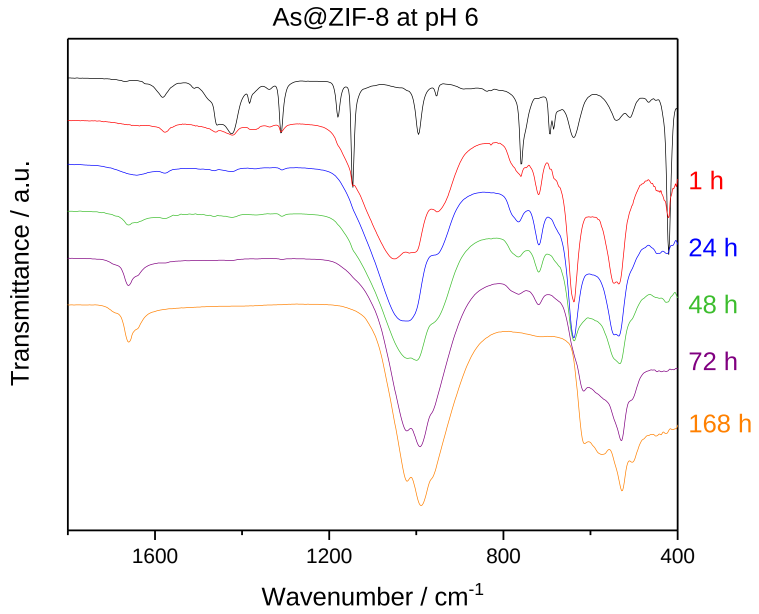

Figure S18. FTIR spectra of As@ZIF-8 (black) and As@ZIF-8 after the arsenic release studies carried out at pH 6 for 1h (red), 24 h (blue), 48 h (green), 72 h (purple) and 168 h (orange) displayed in the area from 1800 to 400 cm^{-1}.

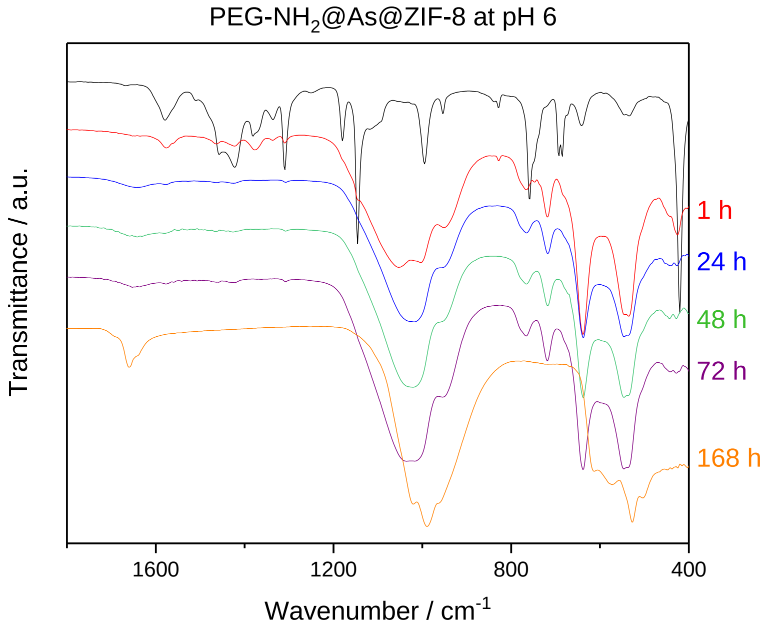

Figure S19. FTIR spectra of PEG-NH_2@As@ZIF-8 (black) and PEG-NH_2@As@ZIF-8 after the arsenic release studies carried out at pH 6 for 1h (red), 24 h (blue), 48 h (green), 72 h (purple) and 168 h (orange) displayed in the area from 1800 to 400 cm^{-1}.

Thermogravimetric analysis:

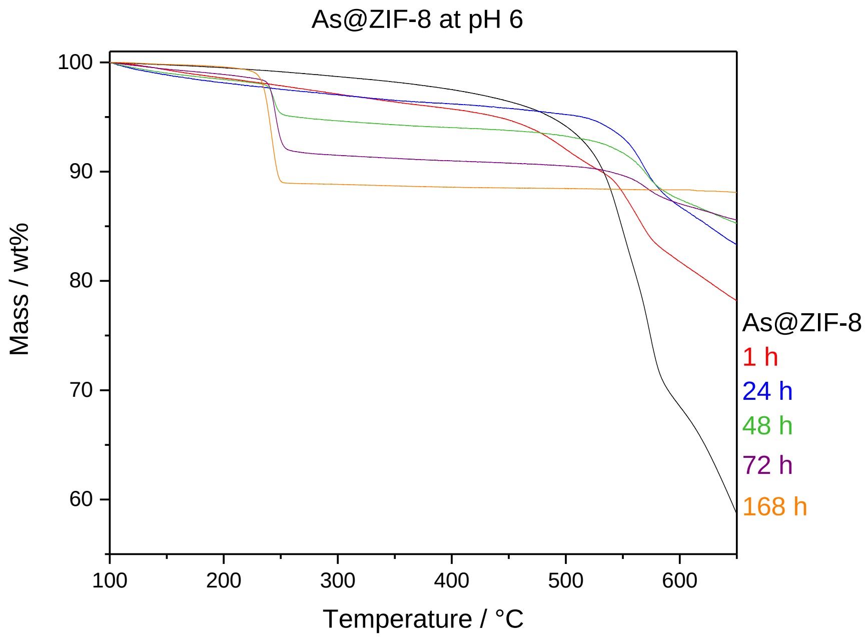

Figure S20. Thermogravimetric analysis of As@ZIF-8 (black) before and after the arsenic release studies carried out at pH 6 for 1 h (red), 24 h (blue), 24 h (green), 72 h (purple) and 168 h (orange) (samples were activated at 100 °C for 3 h and exposed to a flow of nitrogen gas).

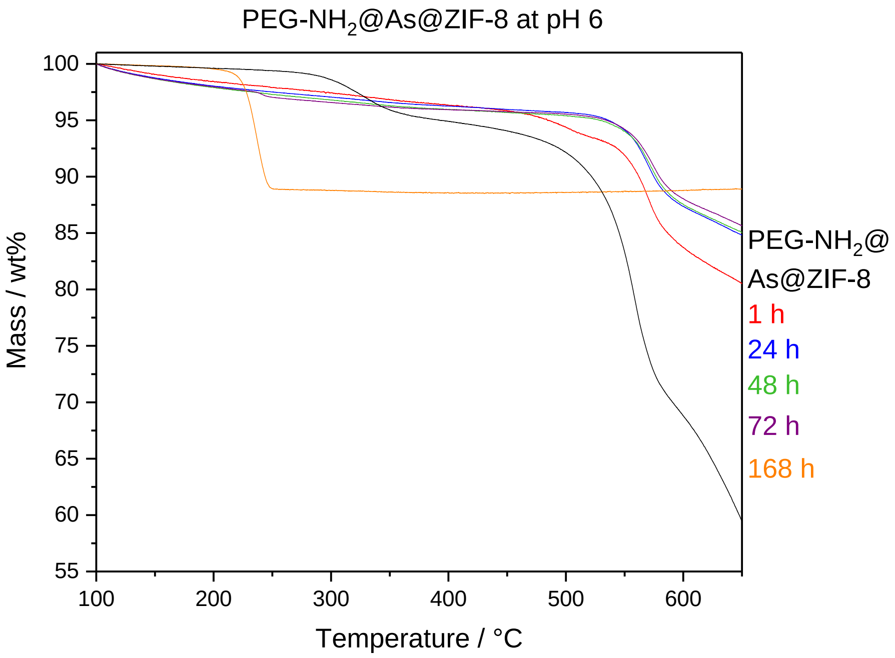

Figure S21. Thermogravimetric analysis of PEG-NH$_2$@As@ZIF-8 (black) before and after the arsenic release studies carried out at pH 6 for 1 h (red), 24 h (blue), 24 h (green), 72 h (purple) and 168 h (orange) (samples were activated at 100 °C for 3 h and exposed to a flow of nitrogen gas).

Cytotoxicity studies

The composition of the nanoparticles of As@ZIF-8 and PEG-NH_2@As@ZIF-8 was determined by ICP-OES (Table 2). The mass for the different samples for the cytotoxicity studies was calculated with respect to a fixed concentration of arsenic (As@ZIF-8 and PEG-NH_2@As@ZIF-8). Corresponding to this result, the equivalent amount of ZIF-8 and organic linker was calculated. For each material, a stock solution was prepared which was further used to prepare the samples of required concentrations.

- Amount of PEG-NH_2@As@ZIF-8 containing As-amount corresponding to 100 µmol of As_2O_3: 202.5 mg
- Amount of As@ZIF-8 containing As-amount corresponding to 100 µmol of As_2O_3: 192.1 mg
- Amount of ZIF-8 which can be loaded with As-amount corresponding to 100 µmol of As_2O_3: 173.8 mg
- Amount of 2-methylimidazole in ZIF-8 which can be loaded with As-amount corresponding to 100 µmol of As_2O_3: 129.4 mg

Table S4. Overview of the calculated amount of each material to prepare sample of the given concentration of As_2O_3 (0.0001 → 100 µM).

Sample	Conc. of As_2O_3 (µM)	PEG-NH_2@ As@ZIF-8 (mg/100 mL)[a]	As@ZIF-8 (mg/100 mL)[b]	ZIF-8 (mg/100 mL)[c]	2-methylimidazole (mg/100 mL)[d]
1	0.0001	0.00002025	0.00001921	0.00001738	0.00001294
2	0.001	0.0002025	0.0001921	0.0001738	0.0001294
3	0.01	0.002025	0.001921	0.001738	0.001294
4	0.1	0.02025	0.01921	0.01738	0.01294
5	1	0.2025	0.1921	0.1738	0.1294
6	10	2.025	1.921	1.738	1.294
7	100	20.25	19.21	17.38	12.94

[a] Amount of PEG-NH_2@As@ZIF-8 containing As-amount corresponding to the given concentration of As_2O_3 (0.0001 → 100 µM).

[b] Amount of As@ZIF-8 containing As-amount corresponding to the given concentration of As_2O_3 (0.0001 → 100 µM).

[c] Amount of ZIF-8 which can be loaded with As-amount corresponding to the given concentration of As_2O_3 (0.0001 → 100 µM).

[d] Amount of 2-methylimidazole in ZIF-8 which can be loaded with As-amount corresponding to the given concentration of As_2O_3 (0.0001 → 100 µM).

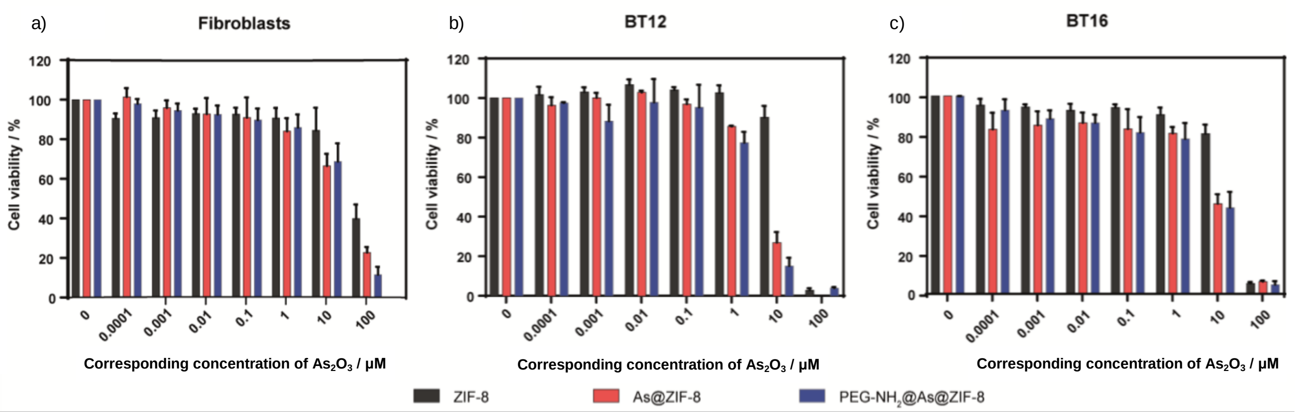

Figure S22. Cell viability of (a) fibroblasts, (b) BT12- and (c) BT16 cells after 24 h of incubation with increasing concentrations of ZIF-8 (black), As@ZIF-8 (red) and PEG-NH_2@As@ZIF-8 (blue). The given concentration corresponds to the concentration of As_2O_3 (0 - 100 µM) which was effectively loaded or could be theoretically loaded (for details see Table S4). Data are presented as mean ± S.E.M (n ≥ 3).

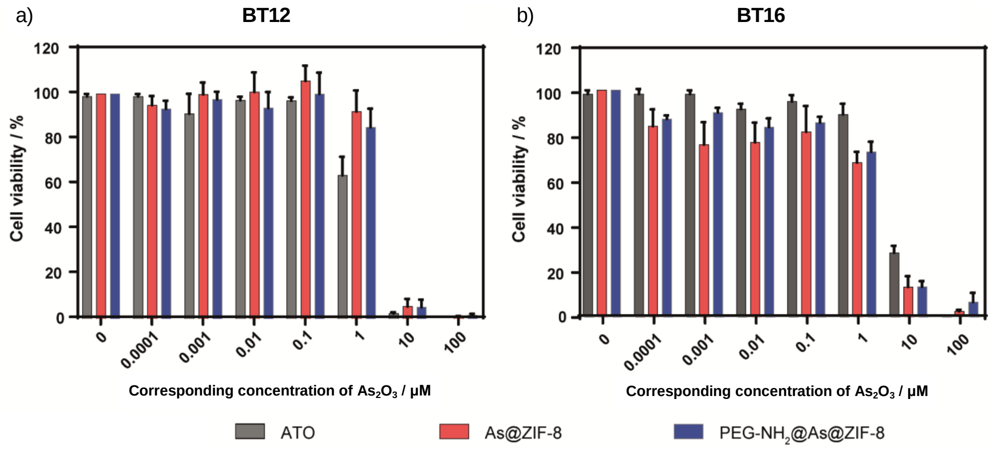

Figure S23. Cell viability of (a) BT12- and (b) BT16 cells after 24 h of incubation with increasing concentrations of ATO (grey), As@ZIF-8 (red) and PEG-NH_2@As@ZIF-8 (blue). The given concentration corresponds to the concentration of As_2O_3 (0 - 100 µM) which was effectively loaded or could be theoretically loaded (for details see Table S4). Data are presented as mean ± S.E.M (n ≥ 3).

References

1 S. J. Clark, M. D. Segall, C. J. Pickard, P. J. Hasnip, M. I. J. Probert, K. Refson and M. C. Payne, *Z. Kristallog. – Cryst. Mater.*, 2005, **220**, 191.

2 E. R. McNellis, J. Meyer and K. Reuter, *Phys. Rev. B*, 2009, **80**, 1.

3 A. Tkatchenko and M. Scheffler, *Phys. Rev. Lett.*, 2009, **102**, 73005.

4 K. Refson, P. R. Tulip and S. J. Clark, *Phys. Rev. B*, 2006, **73**, R4954.

5 T. Bruhn, A. Schaumlöffel, Y. Hemberger and G. Bringmann, *Chirality*, 2013, **25**, 243–249.
